# Awareness of Performance on a Functional Cognitive Performance-Based Assessment Across the Adult Lifespan

**DOI:** 10.3389/fpsyg.2021.753016

**Published:** 2021-11-05

**Authors:** Catherine Arora, Carina Frantz, Joan Toglia

**Affiliations:** ^1^Department of Occupational Therapy, School of Health and Natural Sciences, Mercy College, Dobbs Ferry, NY, United States; ^2^School of Health and Natural Sciences, Mercy College, Dobbs Ferry, NY, United States

**Keywords:** IADL, metacognition, weekly calendar planning activity, cognitive strategies, self-awareness, self-monitoring, cognitive aging

## Abstract

As people age, their cognitive skills and ability to complete complex instrumental activities of daily living often decline in subtle ways. Older adults who are aware of these slight cognitive and functional changes spontaneously adapt and implement strategies to maximize performance. On the other hand, older adults with limited self-awareness are less likely to adjust performance or initiate compensatory strategies as they may not recognize the need to do so. This places them at higher risk of functional decline and loss of independence. Research on awareness of functional performance in healthy adults is, however, limited, and there is a paucity of assessment tools available to address questions of awareness and strategy use in functional tasks. We used the Weekly Calendar Planning Activity (WCPA) – a performance-based assessment of functional cognition including measures of awareness and strategy use – to investigate differences in performance, awareness, and strategy use across the adult lifespan. The WCPA requires examinees to schedule appointments into a weekly calendar while following rules designed to increase task demands. Healthy adults (*n*=342) from ages 18–92 were observed for strategy use and error recognition, while a post-test interview probed participants’ reported strategy use and estimation of accuracy. The discrepancy between participant estimation and actual accuracy provided a measure of online awareness of performance where a larger estimation discrepancy indicated over-estimation of performance. Performance on the WCPA declined across the adult lifespan. Older adults were less likely to use self-monitoring strategies and used less effective strategies overall. Overestimation was associated with use of fewer strategies and lower accuracy in all age groups. Importantly, twice as many older adults overestimated compared to younger adults. Furthermore, the subset of older adults who had good awareness of performance was more likely to use effective strategies, to recognize errors, and achieved accuracy on par with their younger counterparts. Our results emphasize the importance of examining self-awareness of performance and analyzing the strategies used to perform a complex functional task. This information can provide a foundation for early detection of functional decline in aging and for designing interventions to maximize functional independence in aging.

## Introduction

As people age, subtle changes in the ability to perform complex and cognitively demanding instrumental activities of daily living (C-IADL) such as using a calendar, scheduling appointments, taking medication, and managing health and finances are commonly observed (Schmitter-Edgecombe et al., 2011). These changes can reflect age-related declines in cognition, particularly executive function skills such as working memory, inhibitory control, and cognitive flexibility (McAlister and Schmitter-Edgecombe, 2016). Subtle declines in IADL can also be an initial sign of mild cognitive impairment (MCI) or other underlying conditions and may be a marker of those at risk for future decline (Rodakowski et al., 2014; Bezdicek et al., 2020).

Many older adults are acutely aware of these slight decrements in functioning and successfully adjust to them by employing a range of compensatory strategies (Weakley et al., 2019). For example, if a person notices they are having difficulty keeping track of information, they may begin to write more notes, use calendars and lists more frequently, re-organize task materials and take more time to plan ahead. Tomaszewski Farias et al. (2020) found that frequency of spontaneous strategy use in daily life was associated with higher functional independence level, even after accounting for cognition. Their results suggest that strategies can compensate for cognitive decline if a person uses them (Tomaszewski Farias et al., 2018).

Lower levels of awareness have been found to be related to reduced use of strategies and activity modifications (Shaked et al., 2019). If a person is unaware of functional changes in everyday activities, they will be less likely to ask for help, modify tasks or routines or use strategies to improve performance (Shaked et al., 2019). In addition, safety risks and adverse consequences can occur if errors go unrecognized (e.g., misreads nutritional label), or abilities are overestimated (driving collisions). Failure to recognize and adjust to changes in everyday activities places an older person at risk for functional decline, and loss of independence and autonomy (Lee and Dey, 2011; Harty et al., 2013). Self-perceptions of functional abilities influence choices and decisions about everyday activities, including use of strategies or modifications, and the ability to seek help or assistance when needed. Awareness of functional changes and strategy use on C-IADL tasks is therefore critical for safe and healthy aging in place. Research on awareness of performance and strategy use within C-IADL tasks, however, is limited in healthy adults. Additionally, there is a lack of practical tools that can be used to assess these skills.

The Weekly Calendar Planning Activity (WCPA) is a recently developed performance-based functional cognitive assessment that was initially designed for use with clinical populations with impairments in executive functions across the lifespan. It provides an opportunity for analysis of performance including how a person copes with cognitive challenges within an everyday task, including error recognition, strategy use, and self-awareness of performance (Toglia, 2015; Toglia et al., 2017; Jaywant et al., 2021). Further understanding of self-awareness and strategy use of healthy adults within the context of a cognitively demanding functional task, such as the WCPA, is important for optimizing function and tailoring interventions for older adults as well as providing a foundation for interpreting deficits in awareness and strategy use in clinical populations. Further details on the WCPA are described below.

Self-awareness is a broad and complex, multidimensional construct for which there is divergent literature in the fields of social (Morin, 2011; Vess, 2019) and cognitive psychology (Flavell et al., 2002; Dinsmore et al., 2008), neuropsychology (Amanzio et al., 2020) and rehabilitation (Toglia and Kirk, 2000; Ownsworth et al., 2006; Sunderaraman and Cosentino, 2017; Chapman et al., 2020). The conceptualization of self-awareness used within this paper is based on the Dynamic Comprehensive Model of Awareness (DCMA; Toglia and Kirk, 2000). The DCMA draws from interdisciplinary perspectives (rehabilitation, cognitive, and neuropsychology) and has been applied across clinical populations and healthy adults (Schoo et al., 2013; Goverover et al., 2014; Ng et al., 2018; Chen and Toglia, 2019). It distinguishes between offline and online awareness. Offline awareness involves general self-knowledge of strengths and weaknesses that exists outside the context of task performance and is based on accumulated experiences or stored memories (Toglia and Kirk, 2000). It is typically assessed by comparing discrepancies between subjective self-ratings of abilities across different domains (e.g., physical, functional, behavioral, and cognitive) with reports of knowledgeable informants, using a questionnaire or interview, outside the context of engagement in an activity (Dromer et al., 2021).

*On-line awareness of performance* is activated within the context of activity performance and is described broadly to include awareness immediately before, during, or after engagement in a task. Online awareness includes metacognitive skills such as task appraisal (online anticipatory awareness), online error recognition and monitoring as well as the ability to accurately assess one’s performance immediately after a task and recognize discrepancies between expected and actual performance (online emergent awareness; Toglia and Kirk, 2000). Online awareness that emerges with task experiences can influence future task appraisal and performance (Toglia and Kirk, 2000; Ng et al., 2018).

Assessment of online awareness occurs within the context of a specific activity. It may include frequency counts of spontaneous error correction and examination of discrepancies between self-appraisal and actual performance (O’Keeffe et al., 2007). The performance discrepancy method is commonly used within memory list learning tasks (Chudoba and Schmitter-Edgecombe, 2020) and within the context of functional activities as it examines a person’s awareness or perceptions of specific abilities in close proximity to a related task (Goverover et al., 2014; Rotenberg-Shpigelman et al., 2014; Chen and Toglia, 2019; Chudoba and Schmitter-Edgecombe, 2020).

The majority of literature on awareness of functional abilities has relied on offline or questionnaire methods of assessment. Awareness of performance within a task context has potential to provide important information related to everyday functioning. However, very few studies have examined awareness within the context of engagement in C-IADL activities (Chudoba and Schmitter-Edgecombe, 2020), and none have done so across the healthy adult lifespan.

In general, people tend to over-estimate their performance in many domains (Huang et al., 2020; Sunderaraman et al., 2020; Wöstmann et al., 2021); however, the degree of over-estimation is the largest among the poorest performers. This phenomenon, known as the “Dunning-Kruger Effect” (Kruger and Dunning, 2000), has been demonstrated in laboratory studies as well as real-life tasks with college-aged students (e.g., performance on exams or debates; Miller and Geraci, 2011), and healthy adults in workplace settings (Ehrlinger et al., 2008). The effect of age on awareness of complex functional task performance has been studied in some specific contexts. For example, older adults, who overestimate abilities or have a greater discrepancy between self-ratings and those of an expert, also have a higher frequency of unsafe driving behaviors (Huang et al., 2020). Similarly, overestimation of financial abilities has been found to be associated with lower accuracy in financial decision-making (Sunderaraman et al., 2020). It is unclear if overestimation and lower performance in the domains of driving and financial management translate to other functional activities of daily living.

Despite the importance of self-awareness and estimation of abilities in everyday activities, performance-based C-IADL assessments do not typically include measures of self-awareness. C-IADL is commonly assessed using self-reports, informant reports, or performance-based assessments. While self-report provides the person’s perspective, reports of functioning may be considerably overestimated and influenced by personality variables, willingness to acknowledge difficulties, emotional status, social desirability, or decreased self-awareness (Suchy et al., 2010; Buchanan, 2016). Similarly, informant or proxy reports are dependent on observations of others that can be biased and may be overshadowed by the informant’s expectations, stress, or perceived burden (Long et al., 1998). Performance-based C-IADL assessments typically assess competency, independence level, or amount of assistance needed in completing specific C-IADL tasks. Performance-based measures of IADL have been found to differentiate healthy older adults from those with MCI (Puente et al., 2014; Rycroft et al., 2018). However, such assessments often do not consider the person’s awareness of their own performance or how the person goes about performing the task and the strategies used.

The WCPA was designed to include novelty and rule constraints that require a strategic approach and place increased demands on executive function skills. Examinees are required to schedule a list of appointments (10 or 17 appointments depending on the version of the test used) onto a weekly calendar and adhere to rules including avoiding conflicts, keeping a designated day free, ignoring questions from the examiner designed to distract from the task, and keeping track of time. In addition, there are unexpected obstacles in the calendar format; for example, time blocks change from 15min intervals to 30min intervals in the evening portion of the calendar. Some appointments include choice of days and/or times, while others do not, requiring examinees to plan ahead and review the list to avoid conflicts. While entering appointments on a calendar may be a familiar activity, the WCPA is made significantly more challenging by rule constraints, appointment conflicts, and unexpected changes in the WCPA calendar format. The WCPA allows for observation of examinees’ approach on a complex C-IADL task as well as their perceptions of the task. Examinees are observed during the activity for strategy use and error recognition while during a post-task interview examinees are asked to rate the difficulty of the task and estimate their performance accuracy (Toglia, 2015). Demonstrations of the administration of the WCPA are available for further review (Toglia, 2021).

The standard 17-item version of the WCPA distinguishes between healthy controls and people with executive dysfunction including those with attention-deficit hyperactivity disorder (Lahav et al., 2018), epilepsy (Zlotnik et al., 2020), and multiple sclerosis (Goverover et al., 2020). The WCPA allows measurement of awareness in multiple ways (Toglia, 2015). Better awareness of performance in healthy adolescents has been found to be associated with higher accuracy on the 17-item WCPA (Zlotnik and Toglia, 2018). The shorter, 10 item version of the WCPA (WCPA-10) differs from the WCPA-17 only in the number of appointments required to schedule and has an important advantage of taking less time to administer. The WCPA-10 differentiates between healthy adults and individuals’ post-stroke (Jaywant et al., 2021) or with MCI (Lahav and Katz, 2020). Lahav and Katz (2020) also reported declining IADL performance in participants with MCI compared with healthy controls although the correlation between IADL performance and WCPA-10 performance was not reported.

Adult normative data on the WCPA-10 could provide insights into relationships and differences between performance, strategy use, and online awareness on a C-IADL task across healthy adult age groups while at the same time increasing utility of the WCPA-10 as a functional cognitive assessment in older adults or adults with clinical conditions. We sought to examine differences between young, middle-aged, and older adult age groups on the WCPA-10 and in particular awareness of performance and its relationships with accuracy and strategy use across the adult lifespan. Specifically, our questions were: (1) Does WCPA-10 performance (e.g., time, accuracy, efficiency, and adherence to rules) differ across the adult lifespan?; (2) Does strategy use differ across the adult lifespan, both in terms of numbers of strategies and types of strategies?; (3) What is the relationship between strategy use and performance on the WCPA-10?; (4) Do the proportions of people who use self-monitoring strategies, self-recognize errors, and have good awareness of accuracy differ between age groups?; and (5) Within each age group, what is the relationship between awareness of performance and accuracy, self-recognition of errors, and use of strategies?

We hypothesized that performance on the WCPA-10 would decrease sequentially from younger to older adults. We further expected that younger and older age groups would differ in the number and types of strategies used and that younger adults would be more likely to use strategies requiring greater cognitive resources such as grouping or re-organization of information (Lemaire, 2010; Guerrero Sastoque et al., 2019). We hypothesized that awareness of performance, self-recognition of errors, and use of self-monitoring strategies would decrease sequentially from younger to older adults. Finally, we expected that within age groups, there would be an inverse relationship between awareness levels and accuracy, self-recognition of errors, and use of strategies.

## Materials and Methods

### Study Population

Participants (*N*=324), aged 18–92, were recruited *via* convenience sampling from New York City and surrounds. Inclusion criteria included community-dwelling adults who were independent in instrumental activities of daily living and for whom English was their primary language. The Montreal Cognitive Assessment (MoCA) was used as a cognitive screen for participants aged 65 and over. Exclusion criteria included prior diagnosis of a neurological condition (e.g., stroke, traumatic brain injury, Parkinson’s disease, brain tumor, and attention-deficit hyperactivity disorder), prior hospitalization for a psychiatric disorder, or MoCA score less than 24 – a cutoff score thought to minimize false-positive diagnosis of mild cognitive impairment (Carson et al., 2018). In addition, participants were excluded if they scored more than 1.5 standard deviations below the mean on the Patient-Reported Outcomes Measurement Information System (PROMIS) Cognitive Abilities Short-Form Version 2.0, Form 8a (Fieo et al., 2016) which is indicative of a subjective cognitive complaint. Collection of normative data from healthy controls was granted exemption by the Mercy College Institutional Review Board (IRB). Verbal consent was obtained after a consent script was read aloud, and a written copy of the script was provided to each participant.

### Weekly Calendar Planning Activity-10

Participants were administered version A of the WCPA-10 a performance-based assessment of functional cognition by graduate occupational therapy students or occupational therapy practitioners who were trained in task administration and scoring by the senior author. Task observation was done in real-time, and the assessment was scored immediately following the task by the person who administered the test and independently by a second trained research assistant. The WCPA takes approximately 15min from beginning to end including scheduling appointments and answering post-test interview questions. A sample calendar is presented in figure 1 of Jaywant et al. (2021).

*Before the task*, participants were instructed to schedule a list of 10 appointments into a weekly calendar while following five rules including avoiding conflicts in scheduling, ignoring random distracting questions, keeping track of time, avoiding scheduling appointments on a pre-determined day, and stating when they were finished. Participants were told that they would be timed but that accuracy was more important than time.

*During the task*, participants were closely observed for self-recognition of errors (described in the next section) and strategy use – including both the number and types of strategies used. Common strategies used by healthy controls to complete the WCPA include those that (i) enhance attention to salient information (i.e., crossing off entered appointments, using finger to direct attention, or highlighting keywords/features); (ii) organize information (i.e., entering fixed appointments before flexible, rearranging materials, using a written plan, or categorize or organize appointments); (iii) assist with keeping track (e.g., verbal rehearsal, crossing off specified free day, or talking out loud about strategy or plan); and (iv) self-monitor performance (self-check, pausing, and re-reading; Toglia, 2015). These strategies are listed in a checklist on which the examiner records participants’ strategy use.

*Immediately after the task*, a post-test interview was used to probe participants’ perceptions of the task. Participants rated their performance by indicating agreement with statements regarding the WCPA-10 on a four-point Likert scale (1=*agree*, 2=*somewhat agree*, 3=*somewhat disagree*, 4=*disagree*). The statements used were “This task was easy for me,” “I used efficient methods to complete this task,” “I completed this task accurately,” and “I kept track of everything I needed to do.” Participants were also asked to estimate the number of appointments they entered correctly, and what strategies they used to complete the task.

#### WCPA Scores

Scores on the WCPA include the number of appointments (of 10) entered into the calendar and numbers of accurate appointments, rules followed, self-recognized errors as well as planning time (time between beginning the WCPA and entering the first appointment), and total time. The final strategy score indicates the number of strategies recorded on the checklist and any strategies reported by the participant (see post-test interview below) which is not represented on the checklist. To account for participants who choose speed over accuracy, despite the instructions, an efficiency score is calculated as total time (s)/weighted accuracy where weighted accuracy is calculated as the percentage of accurate appointments multiplied by accuracy score (Toglia, 2015). Efficiency scores were only calculated for participants who entered 4 or more accurate appointments (*n*=317), and higher efficiency scores are indicative of lower efficiency (Toglia, 2015).

Concurrent, convergent validity and inter-rater reliability has been demonstrated between the standard version of the WCPA and other assessments of executive function (Weiner et al., 2012; Doherty, 2018; Lahav et al., 2018; Goverover et al., 2020).

#### Measures of Awareness in the WCPA-10

The WCPA-10 includes observation of a participant’s approach to the task and a post-test interview to probe participants’ awareness of task demands and performance as described above. Overall, there are four indicators of awareness in the WCPA-10 including (i) use of self-monitoring strategies including “self-checks” and “pausing and re-reading” as observed by the examiner, (ii) self-recognition of errors during the activity as rated by the examiner, (iii) difference between accuracy score and participant estimate of how many of the 10 appointments they entered correctly, and (iv) participant self-ratings regarding their perceptions of task and performance compared to accuracy score.

##### Use of Self-Monitoring Strategies During the Task

During completion of the WCPA-10, examinees were observed for use of a number of strategies as described above, including the self-monitoring strategies: “self-checks” and “pausing and re-reading.” A participant was recorded as having used the “self-checks” strategy if they spontaneously review their work or double-check appointments. Participants were recorded as “pausing and re-reading” if they recognized a conflicting appointment early in the activity and then stopped to re-read instructions or review the list of appointments before continuing. Use of either of these strategies is an indicator of monitoring during performance.

##### Self-Recognition of Errors During the Task

During completion of the WCPA-10, examinees were observed for self-recognition of errors. Examples of self-recognition of errors include acknowledging an error either verbally or non-verbally (e.g., deep sigh or shaking head) or attempting to correct a mistake (e.g., drawing an arrow to the corrected location). Self-recognized errors were quantified as a proportion of total errors which provides a measure of the aspect of awareness of performance concerning error detection. Only participants who made errors were included in this analysis (*n*=284). Error self-recognition scores are presented as percentages with 0% indicating the participant did not recognize any of their errors and 100% indicating all their errors were self-recognized.

##### Estimation Discrepancy: Difference Between Actual and Estimated Score

Estimation discrepancy is defined as the difference between the actual number of accurate appointments and the person’s estimation of the number of accurate appointments, immediately following the task (post-test interview). A score of zero indicates perfect alignment between participant estimate and their score. A score >0 indicates the participant’s estimate was higher than their actual score (and vice versa for scores less than 0). To simplify interpretation for clinical use, we classified participants into “overestimators” and those “aware” of their performance by dichotomizing based on the median estimation discrepancy of the older adults, defining people who scored above the median as over-estimators and people who scored at or below the median as aware of their performance. We chose to classify adults based on the median estimation discrepancy of older adults since this was more conservative than using the median of the entire cohort. Few participants (*n*=9) under-estimated their performance (between 2 and 3 appointments). Since under-estimation indicates the participant recognized their errors, we included these participants in the “aware” group.

##### Task and Performance Rating After the Task: Subjective Self-Ratings of Performance Compared to Actual Performance

Each participant indicated their agreement with statements regarding the WCPA-10 on a scale of 1–4 as described above. An average self-rating was calculated for each participant. Participants were then dichotomized into those who perceived the task to be easy and their performance to be efficient and accurate (average self-rating ≤2) and those who perceived the task to be difficult and/or their performance to be less than efficient, and inaccurate (average self-rating >2).

These groups were further divided into awareness groups based on the median accuracy score of the older adults, again to keep our estimations conservative. Participants who either (i) rated the task as easy (i.e., average self-rating ≤2) and had an accuracy score ≥7, or (ii) rated the task as difficult (i.e., average self-rating >2) and had an accuracy score <7, were classified as aware of their performance. Participants who rated the task as easy but scored <7 were classified as over-raters. Participants who rated the task as difficult but scored ≥7 (*n*=24) were deemed aware of the task difficulty and included in the “aware” group.

### Statistical Analyses

All statistical analyses were completed using SPSS version 26. All variables of interest deviated from a normal distribution (Shapiro–Wilks test). However, inspection of a frequency histogram with normal curve overlay revealed most variables approximated normality. Therefore, mean and median are presented for all variables except for planning time and efficiency scores which were the most significantly skewed. All comparisons were made using nonparametric statistical tests.

Participants were categorized based on age into younger (18–39years), middle-aged (40–64years), and older (65–92years) groups. *Post hoc*, pairwise comparisons were made if a significant overall effect was identified across the three age groups. All values of *p* were Bonferroni adjusted to account for multiple comparisons (Wright, 1992). Differences in demographic characteristics including gender, race, and education between age groups were examined by Chi-squared analysis. Differences in total accuracy between age groups based on gender, race, and education were determined by the Kruskal–Wallis test.

Performance on the WCPA was compared across the three age groups, for planning time, total time, efficiency score, entered appointments, accurate appointments, rules followed, strategies used, and participant estimated number of accurate appointments, by the Kruskal–Wallis test. In terms of strategy use, Spearman’s correlation was used to determine the association between number of strategies used and total accuracy.

Type of strategies were analyzed in two ways, as (i) *categories of strategies* that fulfill a similar aim (i.e., enhance attention to salient information, organize information, assist with keeping track, and self-monitor performance) and (ii) *individual strategies* (i.e., crossing off entered appointments, using finger to direct attention, highlighting keywords/features, entering fixed appointments before flexible, rearranging materials, using a written plan, categorize or organize appointments, verbal rehearsal, crossing off specified free day, talking out loud about strategy or plan, self-checking, and pausing and re-reading). Our analysis of individual strategies was restricted to strategies used by at least 50% of one age group.

The association of *categories of strategies* used with accuracy scores was analyzed within age groups by the Mann–Whitney *U* test. We then compared the frequency of *individual strategies* used by each age group with a Chi-squared analysis. Finally, the association of *individual strategies* used with accuracy was analyzed within age groups by Mann–Whitney *U* test.

For awareness of performance, differences between the percentage of errors self-recognized during the task were compared across age groups by the Kruskal–Wallis test. Spearman’s correlation was used to measure the association between age and the proportion of self-recognized errors.

Pearson’s correlation was used to measure the association between age and estimation discrepancy. We further examined awareness of performance by examining differences in the magnitude of discrepancy between estimated and actual scores across age groups, using the Kruskal–Wallis test. Since poorer performers have a greater chance of a higher estimation discrepancy simply by chance, we generated random estimation discrepancies for each participant based on their accuracy score. We compared the magnitude of these random estimation discrepancies to the actual estimation discrepancy scores within age groups using the Mann–Whitney *U* test. In addition to examining the estimation discrepancy (raw score), we further explored differences in the proportion of adults classified as “aware” or “over-estimators” within each age group, by Chi-squared analysis.

We next examined differences between subjective self-ratings of performance (e.g., task difficulty) and actual performance, across age groups using Chi-squared analysis. Differences in the proportions of participants across age groups who rated the WCPA as “easy” and scored either above or below the median accuracy score of older adults were determined using Chi-squared analysis.

The association between the two classifications of awareness used (i.e., over-estimators or aware using estimation discrepancy, and over-rater or aware using subjective self-rating of performance) was determined using Spearman’s correlation.

Finally, we examined associations between age groups, estimation discrepancy, percentage of errors self-recognized, accuracy, and number of strategies used by Spearman’s correlation. The differences in accuracy score within age and awareness groups (overestimator vs. aware) were determined by the Kruskal–Wallis test. Differences in the individual types of strategies used between those who overestimated (vs. aware) within the younger, middle, and older age groups were determined by Chi-squared analysis. Differences in self-recognition of errors based on awareness groups within age groups were determined by the Mann–Whitney *U* test.

## Results

### Demographic Characteristics

Most participants were female, Caucasian, and had a college education (Table 1). Demographics did not significantly differ between age groups except for gender, *χ*^2^(2, *N*=324)=5.14, *p*<0.077, and education, *χ*^2^(4, *N*=324)=18.89, *p*<0.001. *Post hoc* analyses revealed a significantly higher proportion of the older adult age group were women (*p*<0.03), and a greater proportion of younger (*p*<0.03) and middle-aged (*p*<0.02) adults had a college education compared to older adults. There was no significant difference in accuracy score based on gender (*U*=13,028, *p*<0.79) or race, *H*(6)=7.9, *p*<0.24; however, participants who completed at least some college (*n*=267) had a significantly higher accuracy score than participants whose highest level of education was high school (*n*=58), *H*(2)=24, *p*<0.001.

**Table 1:** Demographic characteristics of adult participants by age groups.

	Younger	Middle-aged	Older	Adj. p^*^
*n*	106	101	117	
Age (years)				–
Mean (SD)	26.8 (5.4)	51.7 (6.8)	72.6 (6.5)	
Median (IQR)	27 (22–30)	52 (47–57)	71 (68–76)	
Gender, *n* (%)				0.09
Male	48 (45.3)	49 (48.5)	40 (34.2)	
Female	58 (54.7)	52 (51.5)	77 (65.8)	
Race, *n* (%)				0.07
Caucasian	61 (57.6)	64 (63.4)	85 (72.7)	
Black/African American	13 (12.3)	13 (12.9)	9 (7.7)	
Hispanic	22 (20.8)	17 (16.8)	17 (14.5)	
Asian/Pacific Islander	3 (2.8)	7 (6.9)	4 (3.4)	
Other	7 (6.6)	0 (0)	2 (1.8)	
Education, *n* (%)				0.001
High school	8 (7.6)	19 (18.8)	31 (26.5)	
Some college	36 (34)	22 (21.8)	38 (32.5)	
Bachelors degree and above	62 (58.5)	60 (59.4)	48 (41.0)	
MoCA Score, mean (SD)	–	–	26.7 (1.7)	–

* Bonferroni-adjusted value of *p* from Chi-square test.

### Performance on the WCPA-10

There were significant differences across age groups for most WCPA-10 scores (Table 2) including for entered appointments, *H*(2)=20.3, *p*<0.001, accuracy scores, *H*(2)=27, *p*<0.001, and percentage of entered appointments that were accurate, *H*(2)=23.9, *p*<0.001. *Post hoc* analyses revealed older adults entered fewer appointments onto the calendar than younger (*p*<0.001) and middle-aged adults (*p*<0.001), suggesting that older adults missed more appointments on the list – i.e., older adults had a greater number of omission errors. Older adults also had a lower accuracy score than younger (*p*<0.001) and middle-aged adults (*p*<0.004). Importantly, lower accuracy among older adults was not a function of having entered fewer appointments, since the percentage of entered appointments that were accurate was also lower in the older age group compared to younger (*p*<0.001) and middle-aged (*p*<0.01) adults.

**Table 2:** Weekly Calendar Planning Activity (WCPA)-10 scores by age group.

WCPA-10 scores	Younger	Middle-aged	Older	Adj. p^*^
*n*	106	101	117	–
Entered appointments				
Mean (SD)^**^	9.8 (0.5)	9.8 (0.6)	9.5 (0.8)	
Median (IQR)	10 (10–10)	10 (10–10)	10 (9–10)	0.001
Total accuracy				
Mean (SD)	8.1 (1.5)	7.7 (1.7)	6.9 (1.9)	
Median (IQR)	8 (7–9)	8 (7–9)	7 (6–8)	0.001
Entered appointments which were accurate, %				
Mean (SD)	82.6 (13.8)	78.1 (17.2)	72.2 (17.3)	
Median (IQR)	88.9 (77.8–90)	80 (70–90)	70 (60–87.5)	0.001
Rules followed				
Mean (SD)	4.5 (0.7)	4.5 (0.8)	4.2 (0.8)	
Median (IQR)	5 (4–5)	5 (4–5)	4 (4–5)	0.002
Strategies used				
Mean (SD)	6.0 (2.1)	6.3 (2.4)	4.6 (2.2)	
Median (IQR)	6 (5–7)	6 (5–8)	4 (3–6)	0.001
Planning time (s)				
Median (IQR)	49.5 (27–194)	45 (18–132)	34 (16–66)	0.02
Total time (min)				
Mean (SD)	11.4 (5.1)	9.9 (4.8)	9.4 (3.7)	
Median (IQR)	9.8 (7.7–14.8)	8.6 (6.6–11.6)	9.0 (7.0–11.0)	0.07
Efficiency score^***^				
Median (IQR)	98.5 (70.9–137.8)	84.1 (61.6–142.5)	104.8 (74.3–165.4)	0.4
Estimated accuracy				
Mean (SD)	9.2 (1.0)	9.3 (1.0)	8.8 (1.4)	
Median (IQR)	9.5 (9–10)	10 (9–10)	9 (8–10)	0.25

* Bonferroni-adjusted value of *p* from Kruskal–Wallis test.

** Mean (SD) are provided for scores which approached normality.

*** Only includes those with 4 or more correct (*n*=318); higher score indicates lower efficiency.

Significant differences across age groups were also found for rules followed, *H*(2)=17.2, *p*<0.002, total strategies used, *H*(2)=35.7, *p*<0.001, and planning time, *H*(2)=11.9, *p*<0.02. *Post hoc* comparisons showed older adults followed fewer rules than younger (*p*<0.003) and middle-aged adults (*p*<0.001), while older adults took less time to plan than younger adults (*p*<0.002).

There was no difference across age groups in total time to complete the WCPA-10, *H*(2)=9.7, *p*<0.07, or efficiency score, *H*(2)=6.0, *p*<0.4. Of note, the number of appointments that participants estimated they had entered accurately during the post-task interview was also not significantly different between age groups, *H*(2)=7.2, *p*<0.25. Finally, there were no significant differences in performance between younger and middle-aged adults.

### Strategy Use on the WCPA-10

Older adults used fewer strategies overall, *H*(2)=35.7, *p*<0.001, than younger (*post hoc p*<0.001) and middle-aged (*post hoc p*<0.001) adults (Table 2). There was a significant positive correlation between the number of strategies used and accuracy score across the whole cohort combined, *r_s_*(324)=0.37, *p*<0.001.

We next analyzed the association between categories of strategies used and accuracy within age groups. Use of strategies to organize information was associated with higher accuracy in older adults (*U*=2,241, *p*<0.003) and middle-aged adults (*U*=1,586, *p*<0.001). Similarly, older adults who used strategies to self-monitor performance (*U*=1,874, *p*<0.02) had a significantly higher accuracy score than older adults who did not use these strategies. These categories of strategies (i.e., organize information, and self-monitor performance) were not associated with accuracy in younger adults. Strategies that enhance attention or assist with keeping track were not associated with higher accuracy in any age group.

**Figure 1:**
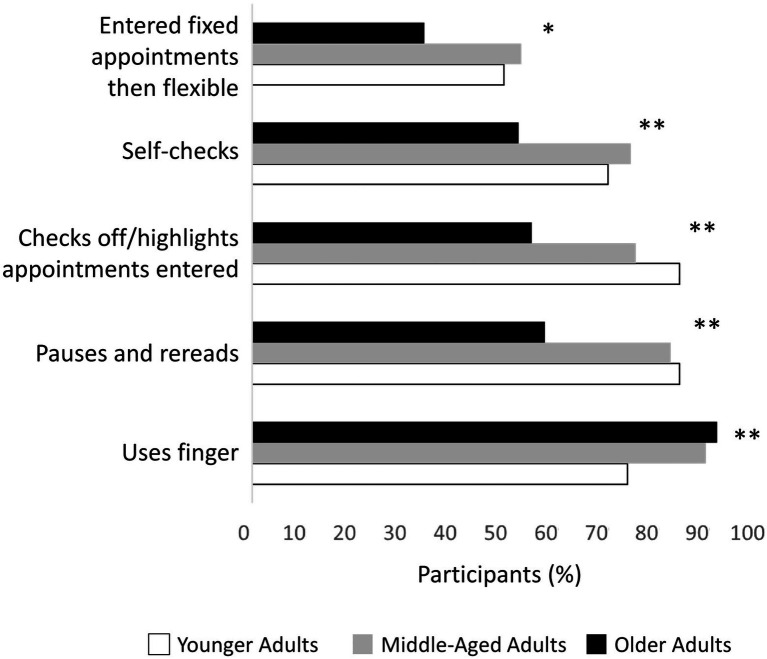
Type and frequency of strategies used across the adult lifespan. Strategies were restricted to those used by 50% of participants in at least one age group. ^*^p < .05; ^**^p < .001.

Five individual strategies were used by at least 50% of one or more age groups, and each strategy was used with differing frequency between age groups, including crossing off entered appointments, *χ*^2^(2, *n*=232)=25.11, *p*<0.001, self-checking, *χ*^2^(2, *n*=213)=13.68, *p*<0.005, pausing and re-reading, *χ*^2^(2, *n*=242)=26.69, *p*<0.001, entering fixed appointments before flexible appointments, *χ*^2^(2, *n*=147)=9.49, *p*<0.04, and using your finger to direct attention, *χ*^2^(2, *n*=278)=16.66, *p*<0.003 (Figure 1).

*Post hoc* comparisons suggest older (*p*<0.001), and middle-aged (*p*<0.01) adults were more likely to use their finger to direct attention than younger adults. Compared to younger adults, older adults were less likely to use the following strategies: crossing off entered appointments (*p*<0.001), self-checking (*p*<0.02), pausing and re-reading (*p*<0.001). Similar differences were found when comparing older and middle-aged adults in *post hoc* analyses. Compared to middle-aged adults, older adults were less likely to cross off entered appointments (*p*<0.004), self-check (*p*<0.002), and pause and re-read (*p*<0.001). Finally, older adults were also less likely than middle aged adults to enter fixed appointments before flexible appointments (*p*<0.01).

Entering fixed appointments first was associated with higher accuracy in middle-aged (*U*=1918, *p*<0.001) and older adults (*U*=2046, *p*<0.003) while crossing off entered appointments was associated with higher accuracy in older adults (*U*=2,319, *p*<0.001). There were no specific strategies associated with accuracy in younger adults after Bonferroni correction.

### Differences in Awareness of Performance on the WCPA-10 Between Age Groups

Younger and middle-aged adults were more likely to monitor their performance by using the “self-checks” and “pausing and re-reading” strategies as described above (Figure 1; Table 3). There was no difference, however, in the proportion of errors that were self-recognized during the task, between age groups, *H*(2)=3.8, *p*<0.15 (Table 3). In addition, there was no association between age and the proportion of errors self-recognized, *r_s_*(322)=0.04, *p*<0.6, suggesting that while younger adults were more likely to monitor their performance during the WCPA-10, this did not translate to increased error recognition.

**Table 3:** Awareness of performance on the WCPA-10 by age groups.

Awareness Measure	Younger	Middle-aged	Older	Adj. p^*^
*n*	106	101	117	
Self-monitoring strategies, *n* (%)				
Self-checking	75 (70.8)	76 (75.2)	73 (62.3)	0.02
Pausing and re-reading	90 (84.9)	84 (83.2)	68 (58.1)	0.001
Errors self-recognized, %				
Mean (SD)	28.9 (37.1)	22.2 (33.6)	29.2 (34.0)	
Median (IQR)	0 (0–50)	0 (0–33)	20 (0–50)	0.5
Estimation discrepancy				
Median (IQR)	1 (0–2)	1 (0.5–2)	2 (1–3)	0.001
Awareness groups^**^, *n* (%)				0.001
Aware	89 (84.0)	78 (77.2)	73 (62.4)	
Over-estimated	17 (16.0)	23 (22.8)	44 (37.6)	
Self-rating, *n* (%)				
*n* ^***^	84	81	90	
Aware	71 (84.5)	67 (82.7)	57 (63.3)	0.001
Over-rated	13 (15.5)	14 (17.3)	33 (36.7)	

* Bonferroni-adjusted value of *p* from Chi-square test except for errors self-recognized and estimation discrepancy which were analyzed using Kruskal–Wallis test.

** Cohort dichotomized based on median estimation discrepancy of older adults.

*** Number of adults who subjectively self-rated the WCPA-10 as “easy.”

There was a positive correlation between age and the difference between estimated and actual scores (estimation discrepancy), *r*(322)=0.24, *p*<0.0001. This suggests that as age increased, the tendency to overestimate one’s abilities also increased. The median estimation discrepancy was significantly different across age groups, *H*(2)=13.8, *p*<0.001, with older adults over-estimating their performance by two appointments at the median compared to only one appointment at the median in younger (*post hoc p*<0.001) and middle-aged (*post hoc* n.s.) adults (Table 3). A data simulation of randomly generated estimation discrepancies based on the accuracy score of each participant indicates that the actual estimation discrepancy seen in each age group is larger than that expected by chance (*p*<0.001).

When participants were dichotomized into awareness groups (based on the median estimation discrepancy of older adults), a greater proportion of older adults were classified as over-estimators [*χ*^2^(2, *N*=324)=14.23, *p*<0.001, Table 3]. *Post hoc* comparison found more than twice as many older adults had an estimation discrepancy above the median than younger adults (*p*<0.001).

Investigation of participant awareness was supplemented *via* analysis of subjective self-ratings of performance on the WCPA-10. Most participants rated the WCPA-10 as “easy” (*n*=255, 79%), and there was no significant difference in self-ratings between younger, middle-aged, or older adults, *χ*^2^(2, *N*=324)=0.37, *p*<0.8. Among participants who rated the task as “easy,” fewer older adults were classified as “Aware” (i.e., scored above the median accuracy score), *χ*^2^(2, *N*=324)=13.42, *p*<0.001 (Table 3). *Post hoc* analysis suggests that older adults tended to over-rate their performance compared to younger (*p*<0.001) and middle-aged (*p*<0.005) adults.

Finally, there was a significant correlation between the two classifications of awareness in our study: that defined by estimation discrepancy and that defined by subjective self-ratings and accuracy, *r_s_*(322)=0.61, *p*<0.001.

### Analysis of Over-Estimators Compared to Aware Participants Within Age Groups

**Figure 2:**
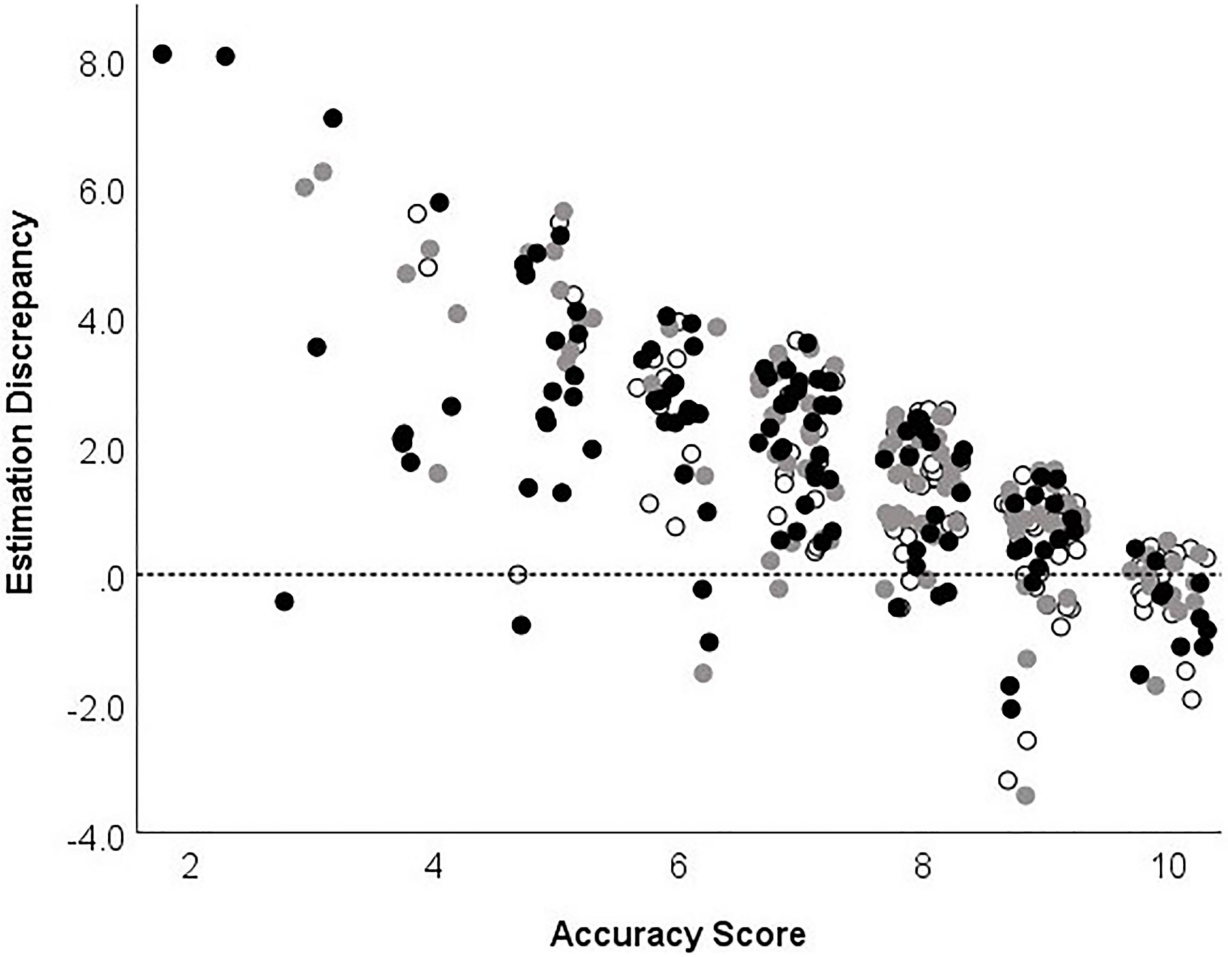
Association between estimation discrepancy and accuracy across the adult lifespan. Dotted line indicates perfect alignment between estimated accuracy and actual accuracy.

**Figure 3:**
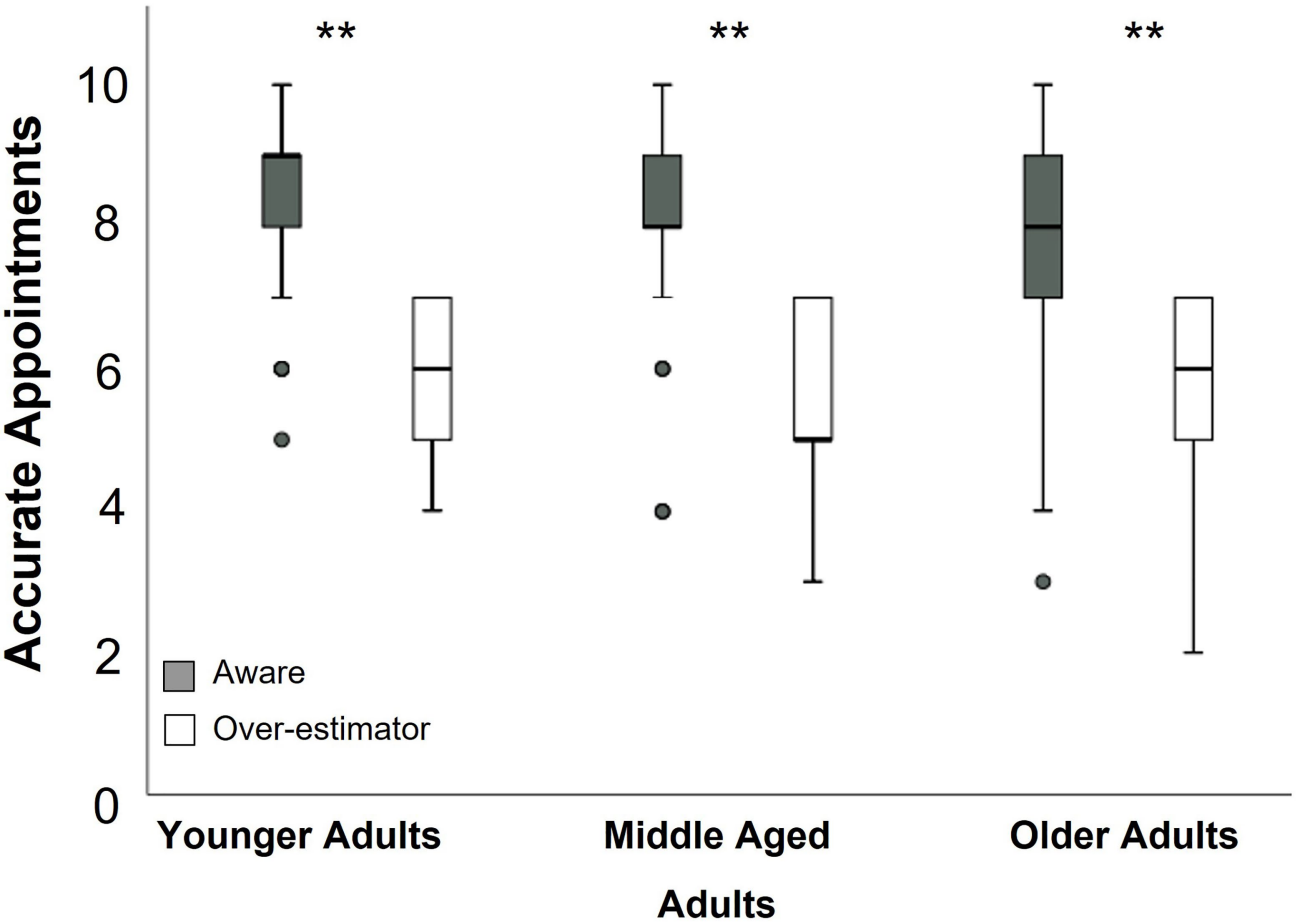
Accuracy of over-estimators compared to aware participants across the adult lifespan. ^**^p<0.001.

Table 4 summarizes, within age groups, the associations between estimation discrepancy, number of strategies used, proportion of errors self-recognized, and accuracy scores. Within younger, middle-aged, and older adults, the difference between estimated accuracy and actual accuracy was significantly, inversely correlated with total accuracy (Table 4). Figure 2 demonstrates the relationship between age group, accuracy score, and estimation discrepancy. In general, estimation discrepancy is higher when accuracy scores are low. At the other end of the spectrum, participants with a high accuracy score tended to provide a more accurate estimation of performance or underestimate their accuracy. The distribution of accuracy scores of participants with good awareness of performance compared to over-estimators is shown in Figure 3. Of note, older adults with good awareness had similar accuracy to younger adults (Figure 3).

**Table 4:** Correlations between estimation discrepancy and accuracy, number of strategies used, and self-recognition of errors in age groups.

	Estimation discrepancy, *r_s_*(*p*)		
	Younger	Middle-aged	Older
*n*	106	101	117
Total accuracy	−0.75 (0.001)	−0.78 (0.001)	−0.68 (0.001)
Strategies used	−0.26 (0.003)	−0.31 (0.004)	−0.22 (0.04)
Errors self-recognized^*^	−0.25 (0.02)	−0.07 (1)	−0.26 (0.008)

* Included only participants who made at least one error: younger: *n*=90; middle-aged: *n*=87; older: *n*=107.

Within age groups, estimation discrepancy was also significantly, inversely associated with the number of strategies used (Table 4). We therefore sought to examine differences in the individual strategies used by adults who were aware of their performance compared to those who over-estimated. A greater proportion of older adults who were aware of their performance (vs. older adults who over-estimated) crossed off entered appointments as an external strategy for keeping track although this was not significant after Bonferroni correction, *χ*^2^(2, *n*=117)=1.75, *p*<0.19. Middle-aged adults who were aware (vs. middle-aged adults who over-estimated) were more likely to enter fixed appointments before flexible appointments, *χ*^2^(2, *n*=101)=12.05, *p*<0.001. Younger adults who had good awareness of their performance (vs. younger adults who over-estimated) were less likely to cross off entered appointments although this was not significant after Bonferroni correction, *χ*^2^(2, *n*=106)=1.34, *p*<0.25.

Finally, within age groups, estimation discrepancy was also significantly, inversely associated with self-recognition of errors (Table 4). Older adults who had good awareness of their performance recognized a median of 50% of their errors compared to older adults who over-estimated their accuracy, who recognized only 18.3% of their errors (*U*=936, *p*<0.04). Overall, adults with better awareness of performance appeared to be more likely to use a higher number of strategies, self-recognize their errors and have greater accuracy.

## Discussion

The aims of our study were to investigate awareness of performance and strategy use on a complex cognitive functional task (WCPA-10) across the healthy adult lifespan. Our results indicate a higher proportion of older adults overestimated their performance and used fewer strategies and that this appeared to be associated with poorer performance on the WCPA-10. Performance patterns observed across young, middle-aged, and older adults on the WCPA-10 are consistent with normative data on the standard 17-item version of the WCPA (Toglia, 2015). Older adults took less time to plan, broke more rules, entered fewer appointments, and were less accurate than younger and middle-aged adults. The WCPA-10 has increased utility over the standard 17 item WCPA because it takes less time to administer. The normative data described in this study will further increase the value of the WCPA-10 as a performance-based C-IADL measure that is easy to administer and yields insight into the examinee’s performance on complex cognitive tasks including awareness of performance and spontaneous use of strategies.

Our results contribute to the growing literature on the importance of self-awareness and metacognitive skills for older adults, within the context of relevant everyday activities (Shaked et al., 2019; Chapman et al., 2020). Our findings demonstrating that older adults with good self-awareness performed on par with younger adults suggest that the ability to accurately appraise one’s own performance may be an important mediator of performance and an important target for intervention. Consistent with the literature on the Dunning–Kruger effect (Kruger and Dunning, 2000), we observed over-estimators in all age groups, and over-estimation was associated with lower accuracy in all age groups. Importantly, however, the proportion of over-estimators in older adults was more than double that in younger adults.

It should be noted, however, that this may have been related to lower accuracy scores among the older group. Since older adults overall performed more poorly than their younger counterparts, there is greater potential for older adults to have a wider discrepancy between their estimated and actual scores. Our results, however, were further supported by our findings related to subjective self-ratings of the task and performance. Approximately 40% of the older adults who rated the task as “easy” scored below the median compared to only 16% of younger adults who rated the task as “easy.” This suggests that older adults may have less accurate perceptions of task difficulty and less accurate assessments of performance than younger adults.

Future research is needed to more clearly delineate discrepancies between one’s estimated and actual performance from cognitive ability level. Nevertheless, the combination of poorer performance and less accurate appraisal of performance has significant consequences for functional independence and healthy aging of older adults given the other factors impacting functional independence in older adults including cognitive and physical decline. However, those adults who accurately appraised performance surprisingly performed as well as other age groups.

Another unexpected finding of our work was that self-recognition of errors *during* the activity was not significantly different among age groups. This is particularly surprising given that there were observed differences in frequency of self-monitoring strategies such as self-checking, between younger and older age groups. It appears that although older adults recognized errors as well as younger adults, they may not have appreciated the significance of the errors or they might not have been able to translate this information into effective task strategies. This is similar to observations by Guerrero Sastoque et al. (2019) who observed that older adults were unable to use error monitoring information to adjust performance. We did observe, however, that older adults with high awareness recognized a greater proportion of errors during the task, suggesting that good awareness may be particularly important for older adults. It should be noted, however, that interpretation of this data is confounded by both the low number of errors overall in younger and middle-aged adults and the large variability in rates of error detection. Further studies are required to discern the utility of the WCPA-10 for quantifying error detection and to clarify the association, or lack thereof, with age.

The vast literature on performance monitoring during memory and learning tasks is somewhat conflicting (Daniels et al., 2009; Hertzog and Dunlosky, 2011; Siegel and Castel, 2019). Some studies have found that older adults overestimated their ability to remember word pairs and over-estimated their perceptual abilities (Palmer et al., 2014; Siegel and Castel, 2019). Additional studies have reported diminished error awareness in older adults, supported by evidence of age-related deficits in neural response to errors, for tasks requiring close performance monitoring (Harty et al., 2017; Sim et al., 2020). Yet, other studies indicate that adults retain the ability to monitor their learning throughout their lifespan (Hertzog et al., 2010; Berry et al., 2013; Sanders and Berry, 2021) which seems in contrast to our findings. This inconsistency is likely due to inherent differences in the cognitive skills required and the nature of the tasks used. We investigated adults’ perception of performance following completion of a cognitively demanding C-IADL task, while the literature on performance assessment during learning is largely generated using recall of word pairs (Hertzog and Dunlosky, 2011; Berry et al., 2013; Zakrzewski, 2021). Recollection of word pairs is a relatively simple task, whereas the WCPA-10 is a complex task requiring multi-tasking, planning, close performance monitoring, and selective attention. Recollection of word pairs is also a non-contextual activity, whereas the WCPA-10 more closely resembles an everyday task. Finally, an item-by-item assessment of performance as required in studies on recall of word-pairs allows the opportunity for refining judgments based on prior items and does not require the participant to keep track of their performance on the task as a whole, unlike a functional task like the WCPA-10. Our study serves to complement the existing literature by expanding the understanding of awareness of task performance in older adults using an instrument that more closely approximates real-life situations. Understanding adults’ perceptions of performance immediately after a task is also important as it is this assessment that can update metacognitive knowledge and influence future performance (Toglia and Kirk, 2000; Ng et al., 2018; Chudoba and Schmitter-Edgecombe, 2020).

An interesting trend from the studies on learning is that monitoring is more effective for unrelated word pairs than related word pairs, which is somewhat counterintuitive (Hertzog et al., 2010). It is possible that related word pairs gave participants a false sense of confidence in their ability to subsequently recall word pairs. By extension, it is possible that common tasks, such as filling in a weekly calendar or other routine tasks of daily life, may be more susceptible to metacognitive errors than truly novel tasks. This would have significant implications for cognitively demanding IADLs required to maintain independence in older age. It is likely that a high proportion of IADLs in older age resembles IADLs that a person has completed their entire adult life. The familiarity of their daily tasks may render older adults at greater risk of absent or ineffective monitoring. Further studies are required to assess the impact of task familiarity on awareness of performance.

Self-awareness affects how individuals appraise a situation and implement strategies that might be utilized to improve performance (Shaked et al., 2019; Scarampi and Gilbert, 2021). In terms of the number of strategies used, our results indicate that people with lower awareness of their performance also utilized fewer strategies and were less accurate. The association between low awareness, reduced strategy use, and low accuracy was most apparent in older adults. This is important given that older adults are the most susceptible to the loss of functional independence and autonomy that would result from a less strategic, and accurate performance on C-IADLs. In our study, older adults who used self-monitoring strategies also had higher accuracy further suggesting a possible link between online awareness of performance and strategy use among older adults.

Overall, older adults used fewer strategies than their younger counterparts. This is consistent with age-related differences in strategy repertoire that have been observed by other researchers (Lemaire, 2010). We also found that awareness in older adults was positively associated with the number of strategies used. In addition to a more limited strategy repertoire, we also observed differences in the type of strategies used. Older adults were more likely to use external strategies such as finger-pointing to direct and focus attention during the task. This strategy is more superficial and places fewer demands on cognitive resources (Bouazzaoui et al., 2010; Guerrero Sastoque et al., 2019). In comparison, younger and middle-aged adults used more self-monitoring and organizational strategies. The latter strategies involving placing fixed appointments first puts greater demands on effortful processing and cognitive resources by requiring the person to self-initiate review of the list, compare and contrast items, recognize patterns, identify priorities, and reorganize information.

It was surprising that the majority of older adults were also less likely to use effective external strategies such as checking off entered appointments to assist with keeping track of appointments. Use of this strategy has the potential to reduce working memory load and decrease omission errors; however, many older adults did not initiate using it. This finding is similar to studies by Hertzog et al. (2019) who found suboptimal use of external memory strategies in older adults and that of Scarampi and Gilbert (2021) who reported that many older adults did not take advantage of using an external strategic reminder even though it was permitted. The latter authors postulated that older adults were overconfident in their unaided abilities and did not use strategies that could reduce cognitive load, even when they had knowledge that these strategies could aid memory. Importantly, we found that older adults with good awareness of performance were more likely to check off entered appointments, suggesting that good self-awareness may potentially influence effective strategy choice.

Why some older adults choose less effective strategies is a pressing question for improving functional performance of aging adults. Our data indicate that the proportion of older adults who make effective strategy choices is much lower than younger adults. Differences in the type of strategies used by older adults are consistent with the previous literature (Lemaire, 2017). One hypothesis is that limitations in processing resources reduce the ability to use more effortful strategies that involve deeper encoding or rely on self-initiated internal cognitive operations. This is supported by findings of decreased use of internal strategies by older adults across different studies (Bouazzaoui et al., 2010) and could explain the choice of more superficial strategies we observed in older adults.

Others have postulated that decreases in effective strategy choices in older age are mediated by cognitive aging particularly in executive functioning skills such as cognitive flexibility and inhibition (Hodzik and Lemaire, 2011). Older adults appear to have difficulty adjusting performance to task challenges. They fail to generate, initiate, or switch task strategies even when they recognize obstacles or performance errors. Instead, they continue to approach the task in the same inflexible way, despite recognition of difficulties. This is further supported by additional studies that have reported older adults delayed switching to more advantageous strategies during tasks, compared to younger adults (and persisted in use of inefficient strategies for a longer time; Touron and Hertzog, 2004; Seaman et al., 2015).

At initial glance, the WCPA appears easy. Many people begin the task with the plan to follow the appointment list in order. Once the person begins the task, they soon encounter conflicts and challenges in keeping track of all information needed. Younger adults were observed to switch plans and strategies as challenges were encountered. In contrast, many older adults commented that the task was harder than they initially thought, but they continued to stick with their initial plan of following the list in order, without checking off items, even though it was apparent that their method was not working.

Alternatively, it may be that older adults do not fully recognize task challenges or the need to use different strategies. For example, Guerrero Sastoque et al. (2019) summarized several studies that have found that older adults are less able to update metacognitive knowledge based on task experiences. It appears that older adults may not be aware of how different strategies or methods they used during performance contributed to task outcomes (Lemaire, 2017; Guerrero Sastoque et al., 2019). This is supported by our observation that the majority of older participants rated the task as “easy” but had lower accuracy and used less effective strategies.

Cognitive training to remediate impaired executive functions is modestly successful within the context of the training activity but has less of an effect on far transfer, including to everyday functioning (Basak et al., 2020; Hertzog et al., 2021). Metacognitive awareness, and strategy use, however, have been shown to be modifiable within older adults and transferable to different contexts (Dunlosky et al., 2003; Levine et al., 2007; Cavallini et al., 2010) and is the basis for a recent intervention approach to address the impact of memory deficits on daily life (Hertzog et al., 2021). Similarly, in cognitive rehabilitation with people with acquired brain injury, metacognitive strategy approaches have been emphasized (Jaywant et al., 2021; Toglia and Foster, 2021).

The trends in the type of strategies used in this study provide insight into potentially effective interventions. For example, checking off items on a list is a strategy that could easily be implemented into the many activities of daily living which involve keeping track of lists to mitigate omission errors. Similarly, use of strategies to organize information was associated with higher accuracy in older adults and could be included in strategy training for everyday complex tasks. Older adults may further benefit from strategies to pace themselves during complex tasks and use external aides effectively to reduce their cognitive load (Guerrero Sastoque et al., 2019). The normative data on strategy use presented in this study support further research in metacognitive and strategy training programs centered around self-monitoring, and self-regulation, organizing, and keeping track of information during everyday complex tasks. Data presented here have the potential to inform healthy aging programs such as that reported by Levine et al. (2007) and are consistent with the self-regulatory approach described by Hertzog et al. (2021).

### Limitations

Participants were recruited *via* convenience sampling and were predominantly Caucasian with a college education which reduces generalizability to the wider population. Our interpretation was limited by the lack of comprehensive neuropsychological data on participants to draw more detailed conclusions about overall cognitive function including the underpinnings of executive functioning and awareness deficits in healthy individuals. The number of older adults with high accuracy scores was also limited, making it difficult to definitely separate ability from estimation discrepancy. We were unable to fully investigate the confounding effect of education on the relationship between age and accuracy due to the small number of participants without a college education.

## Conclusion

Our normative data on performance, awareness, and strategy use across the adult lifespan, on the WCPA-10, add to the utility of this brief functional cognitive tool for identifying possible C-IADL and awareness deficits that might otherwise go unnoticed. Older adults with decreased self-awareness are likely to under-report functional difficulties to others and subtle decline may not be noticed by friends or relatives (Steward et al., 2020). Therefore, a brief performance-based assessment such as the WCPA might be useful in triggering referrals for further assessment and intervention.

Our results suggest that lower-performing adults tend to overestimate their performance and use fewer strategies overall, including self-monitoring strategies, regardless of age. A larger proportion of older adults (more than twice that of younger and middle-aged adults) appear to overestimate or fail to accurately appraise their performance and choose less effective and fewer strategies. This has significant consequences for maintaining functional independence. Conversely, older adults with good error recognition, monitoring strategies, and task appraisal achieved accuracy scores that were similar to younger adults. Our results need to be confirmed with a larger, more representative sample; however, they highlight the importance of examining self-appraisal of task performance and strategy use in older adults. They also suggest that intervention focused on task appraisal, self-monitoring, self-regulation, and strategy use may hold promise for compensating for age-related cognitive changes and optimizing functional independence and is an important direction for future research.

## Data Availability Statement

The raw data supporting the conclusions of this article will be made available by the authors, without undue reservation.

## Ethics Statement

The studies involving human participants were reviewed and approved by Mercy College Institutional Review Board. Written informed consent for participation was not required for this study in accordance with the national legislation and the institutional requirements.

## Author Contributions

CA completed formal data analyses and wrote the initial draft of the paper. CF contributed to data collection, literature review, editing, and review of paper, and completed initial data analysis. JT contributed to conceptualization, methodology, supervision of project, literature review, writing and editing of paper, and project administration. All authors contributed to the article and approved the submitted version.

## Conflict of Interest

JT is author of the Weekly Calendar Planning Activity, published by AOTA Press and receives royalties for this publication.

The remaining authors declare that the research was conducted in the absence of any commercial or financial relationships that could be construed as a potential conflict of interest.

## Publisher’s Note

All claims expressed in this article are solely those of the authors and do not necessarily represent those of their affiliated organizations, or those of the publisher, the editors and the reviewers. Any product that may be evaluated in this article, or claim that may be made by its manufacturer, is not guaranteed or endorsed by the publisher.
